# FEASIBILITY OF A COMMUNITY-BASED NAVIGATOR SUPPORT PROGRAM FOR CARE PARTNERS OF SERIOUSLY ILL VETERANS

**DOI:** 10.1093/geroni/igad104.1523

**Published:** 2023-12-21

**Authors:** Nathan Boucher, Hollis Weidenbacher, Madeleine Eldridge, Kimberly Johnson, Courtney Van Houtven, Karen Steinhauser, Kelli Allen

**Affiliations:** Veterans Health Administration/Duke University, Durham, North Carolina, United States; Durham VA Health care System, Durham, North Carolina, United States; Durham VA Health care System, Durham, North Carolina, United States; Duke University, Durham, North Carolina, United States; Duke University School of Medicine, Durham, North Carolina, United States; Duke/Durham VA, Durham, North Carolina, United States; Durham VA Health care System, Durham, North Carolina, United States

## Abstract

We examined the feasibility and satisfaction of a short-term Veterans Health Administration (VHA)-based care partner support intervention delivered by trained navigators during COVID-19 through a single-arm interventional cohort feasibility study of a telephone-based care partner navigation intervention. Navigators (n=9) received the standard state curriculum online from a local community health worker training program plus an additional eight weeks of online role-specific training by the study team. Subsequently, navigators contacted care partners (n=30) of seriously ill veterans weekly via telephone over a 12-week period to assess social and practical needs, and connect care partners to appropriate community-based or VHA resources. All navigators (9 female; 1 male) and 17% of care partners were African-American (83% were Caucasian or other). Care partners were 90% female and 10% male; age range 41 to 80. In this telephone-based support program delivered to care partners by standard-trained navigators across 12 counties, care partners were overall very satisfied (x̄=4.69 out of 5, sd =.77) with their navigator’s responsiveness and social support throughout the 12-week course of the study. Navigators’ confidence increased over time (x̄=8.2 out of 10 (sd=1.14) to x̄=8.78 (sd=.97)), while their self-assessed effectiveness ratings lowered over time as they encountered real-life scenarios (x̄=8.5 (sd=1.43) to x̄=7.89 (sd=.93)). Navigators documented successful connections to services in the VHA and in care partners’ communities. Using remote navigators with minimal but standard training is a feasible and acceptable model of care partner support.

